# Pitfalls in Electrocardiographic Diagnosis of Acute Coronary Syndrome in Low-Risk Chest Pain

**DOI:** 10.5811/westjem.2017.1.32699

**Published:** 2017-04-17

**Authors:** Semhar Z. Tewelde, Amal Mattu, William J. Brady

**Affiliations:** * University of Maryland School of Medicine, Department of Emergency Medicine, Baltimore, Maryland; † University of Virginia School of Medicine, Department of Emergency Medicine, Charlottesville, Virginia

## Abstract

Less than half of patients with a chest pain history indicative of acute coronary syndrome have a diagnostic electrocardiogram (ECG) on initial presentation to the emergency department. The physician must dissect the ECG for elusive, but perilous, characteristics that are often missed by machine analysis. ST depression is interpreted and often suggestive of ischemia; however, when exclusive to leads V1–V3 with concomitant tall R waves and upright T waves, a posterior infarction should first and foremost be suspected. Likewise, diffuse ST depression with elevation in aVR should raise concern for left main- or triple-vessel disease and, as with the aforementioned, these ECG findings are grounds for acute reperfusion therapy. Even in isolation, certain electrocardiographic findings can suggest danger. Such is true of the lone T-wave inversion in aVL, known to precede an inferior myocardial infarction. Similarly, something as ordinary as an upright and tall T wave or a biphasic T wave can be the only marker of ischemia. ECG abnormalities, however subtle, should give pause and merit careful inspection since misinterpretation occurs in 20–40% of misdiagnosed myocardial infarctions.

## INTRODUCTION

The chief complaint of “chest pain” causes consternation for countless healthcare providers. Although it accounts for more than eight million emergency department (ED) visits annually, only a fraction will actually have an acute coronary syndrome (ACS). 1,2 Nevertheless, the possibility of impending cardiac death is worrisome for both the patient and provider alike. In the ED we are challenged with identifying those who are at the lowest risk for major adverse cardiac events and safely discharging this subset home. Disposition is aimed at preventing unnecessary hospital admissions and subsequent downstream testing that can be both harmful and costly. Patients who are suitable for a low-risk evaluation should have no hemodynamic or electrical derangements (i.e., dysrhythmias), a normal or near-normal electrocardiogram (ECG), and negative cardiac biomarkers.^2^ They should also be screened for other life-threatening non-cardiac causes of chest pain.^2^ Thereafter, their symptomatology, risk factors (e.g., diabetes, hyperlipidemia, hypertension) and personal plus family history (e.g., myocardial ischemia, infarction, revascularization) are measured, frequently using a clinical risk-stratification tool (e.g., HEART Score).^2^–^7^ These scoring systems, however, are outside the scope of this article and will be discussed in another article as part of this three-part series. Ultimately those who are low score are considered at minimal risk for ACS based on current data.^2^,^3^,^6^,^7^

Studies seeking to identify which aspect is most significant in the chest pain evaluation have concluded that both ECG and history of present illness (HPI) are pivotal, but imperfect.^4^–^7^ A HPI highlighting exertional chest pain, diaphoresis, vomiting, or a clutching/pressure quality with radiation is “classic” and places the patient at high risk for acute myocardial infarction (AMI), but is not diagnostic.^6^,^7^ In fact studies have shown that even low-risk descriptors, believed to be “atypical” (e.g., sharp, pleuritic, reproducible), are seen in patients with AMI; hence, such narratives should not be negated.^6^,^7^ Moreover, regarding certain populations (i.e., the elderly, women, diabetics), “classic” symptoms are infrequent and a poor determinant in distinguishing between cardiac and noncardiac causes of chest pain,^6^,^7^ leaving the ECG as the other reliable piece of evidence in the evaluation and stratification of patients. Healthcare providers must take care not to dismiss non-diagnostic and subtle ECG findings as normal or irrelevant. Such misclassification can have fatal consequences.

### Nondiagnostic ECG

On ED presentation, fewer than half of patients with a clinical history reminiscent of ACS will have a truly diagnostic ECG.^7^–^10^ The other half will have (1) signs of ischemia, (2) nonspecific ST segment and T-wave (NSSTTW) changes, or (3) a completely normal ECG.^7^–^10^ Disposition of those with either ischemia (i.e., admission) or a truly normal ECG (i.e., risk stratification + cardiac biomarker) is becoming fairly standardized and well defined; but those with NSSTTW changes, defined as ≤1 mm ST elevation or depression with or without reciprocal changes, are more challenging. 8 Although current evidence demonstrates an unchanged overall miss rate in AMI (~2%), what remains clear is that “some proportion of those missed are primarily the result of failure by the emergency physician to detect subtle ST-segment elevation.”^11^ Therefore, however minuscule (≤1mm ST elevation) NSSTW findings should give pause since they may herald an event. Ischemia can be exhibited in several ways, most commonly T-wave inversion (TWI) or ST depression (STD). These two findings are not equivalent. Patients with STD are known to have a poorer prognosis.^8^–^10^ Likewise, patients with NSSTTW changes are more likely than those with a normal ECG to be transferred from observation to an inpatient unit and have a higher likelihood of developing an infarction.^8^–^10^ If an initial ECG is nondiagnostic, NSSTW serial tracings should be obtained to assess for further evolution. 8–10 The ECG is a cornerstone in identification of AMI, and scrutiny for elusive characteristics decreases its likelihood.

### The Forgotten Lead (Figure 1)

Typically, when STD is identified, ischemia becomes the first, second, and third diagnoses considered. Serial cardiac biomarkers are obtained and anticoagulation is initiated. In the following scenario, infarction, not ischemia, should be considered first. Elevation in lead aVR with concomitant diffuse STD has been found in association with diffuse subendocardial ischemia and infarction of the basal septum.^12^ Considered the “forgotten lead,” aVR is frequently ignored and was thought to have no relevance, but its importance has recently become appreciated. In 2013 the Guidelines for Management of ST-elevation Myocardial Infarction (STEMI) issued by the American College of Cardiology Foundation/American Heart Association added multi-lead STD with coexistent ST-elevation in aVR as an indication for acute reperfusion therapy.^13^ This electrocardiographic finding has been observed in patients with left main, proximal left anterior descending, and triple vessel disease.^14^ Controversy in the literature does exist as to whether elevation in aVR is indicative of complete or rather sub-occlusive coronary artery disease.^19^–^20^ Thus far, studies have been small, retrospective, and heterogeneous in defining the type of occlusion, collateral circulation, ischemic conditioning, and various other factors. Irrespective elevation in aVR with reciprocal diffuse depressions warrants early aggressive therapy and should not be mistaken as non-specific. Tachycardia, cardioversion, and cardiopulmonary resuscitation all also can cause diffuse STD that resolves over time with normalization of the heart rate, as witnessed with serial ECGs. These unique circumstances should be remembered so as not to be confused with AMI.

### Posterior AMI (Figure 2)

Another ECG finding that is often mistaken for ischemia when infarction should be considered involves the posterior myocardium. A small percentage of posterior infarcts (~5%) occur in isolation and produce only STD, specifically in leads V1–V3, but the majority of them occur in conjunction with an inferior or lateral infarct, so ST elevations are evident. 21–23 Tall R waves and upright T waves are also characteristically seen in those leads.^21^–^23^ The STD cues many clinicians to diagnose ischemia without considering infarct. Isolated posterior AMI is the most common infarct pattern that is mistaken for ischemia, even though it has been recognized for many years to be secondary to transmural posterior injury. 21 When doubtful regarding infarct versus ischemia, a posterior ECG should be obtained by placing leads V4–6 in the left scapular region. ST elevation of only 0.5 mm in any one lead is diagnostic.^22^, ^24^ Despite the relatively small myocardial involvement with posterior AMI, its clinical sequela is far from inconsequential. It results in moderate to severe mitral regurgitation, an independent predictor of long-term heart failure and infarct-related mortality, in up to one third of patients.^25^

### Inferior AMI (Figure 3)

When electrocardiographic findings are isolated in a single lead, they are frequently placed into the normal or NSSTW category. But even in isolation, certain findings should be considered a forewarning. To many physicians, a lone TWI in aVL would be considered insignificant; however, a number of studies have demonstrated the importance of aVL T-wave changes in recognition of right ventricular involvement, specifically its association with an imminent inferior AMI.^26^–^28^ T-wave changes, especially in lead aVL, have not been emphasized and are not well recognized across all specialties. The accumulating evidence with regard to TWI in aVL indicates that it should not be considered normal or nonspecific despite its isolation.^29^

### Ischemia

**Figure 4:**
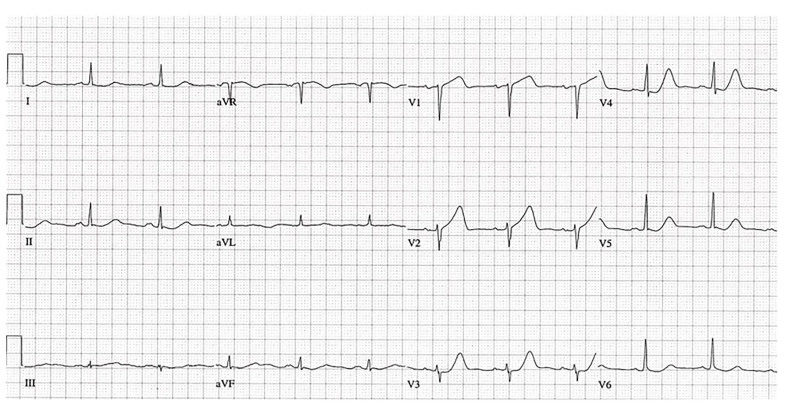
Tall T wave V1. Broad upright T wave V1>V6 with subtle septal (V1–V2) ST elevation and anterolateral (V4–V6, I) ST depression.

In most people, lead V1 looks akin to aVR because the main vector of ventricular depolarization is going away from both leads. During normal depolarization the QRS vector rotates from rightward to left corresponding to deep S waves in the right precordial leads (V1–2) and larger R waves in the left precordial leads (V5–6). The midprecordial leads (V3–4) typically show equal R and S waves; hence, it’s called the transitional zone. The direction of the T wave in V1 depends on how much the vector is oriented anteriorly; it may be upright or inverted, but it’s expected to be upright throughout the rest of the precordium. Although an upright T wave in V1 is considered a “normal variant,” caution should be taken when the T wave is both upright and large. Specifically when it’s taller than the T wave in lead V6 it is referred to as loss of precordial T-wave balance (Figure 4).^30^ This scenario portends a high likelihood of coronary artery disease and, when new, should raise concern about ischemia.^31^–^34^

**Figure 5:**
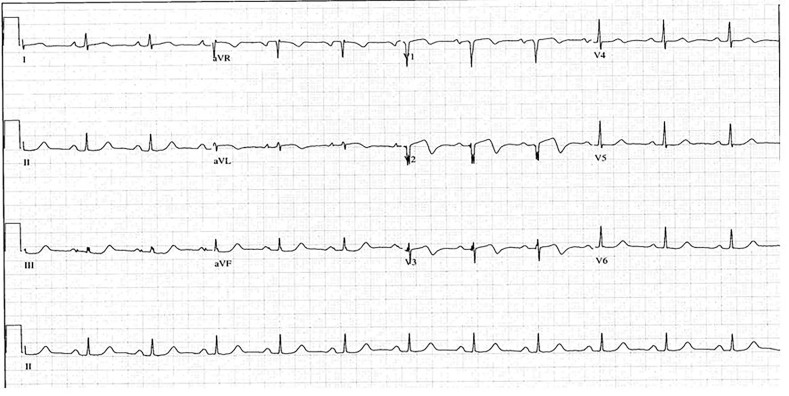
Wellens’ syndrome. Biphasic T waves V2–V3 with minimal ST elevation.

Another troublesome finding is a biphasic T wave. An initial positive deflection followed by terminal negativity in leads V2 and V3 is highly specific for subacute stenosis of the left anterior descending artery.^35^, ^36^ This pattern is indicative of Wellens’ syndrome (Figure 5). It was first described by Gerson and colleagues in 1980 as an inverted U-wave pattern^37^–^38^ and then further delineated by De Zwaan and associates in 1982. It consists of characteristic electrocardiographic findings suggesting severe stenosis of the proximal left anterior descending artery, which, in most untreated patients, develops into an anterior AMI within days to weeks. The syndrome has two forms. Type A, the more common form (occurring in ~75% of cases), is characterized by deeply inverted T waves in V2 and V3.^35^–^36^ Type B, characterized by biphasic T waves in V2 and V3, occurs in ~25% of cases. 35–36 When Wellens’ syndrome is suspected, urgent activation of cardiac catheterization resources is recommended.^39^–^41^ Provocative testing is not endorsed, since increasing cardiac demand in a patient with a highly stenosed left anterior descending artery could lead to complete occlusion, resulting in dysrhythmia and even cardiac arrest.^39^–^41^

## CONCLUSION

Despite growing sophistication in computer-based analysis of ECGs, subtleties are often missed by these devices. STD read as ischemia or isolated TWI and biphasic T waves called normal or nonspecific respectively. Practitioners should not be falsely reassured since we know many patients will present this way yet go on to have acute coronary syndrome. The astute physician will recognize that a nonspecific or nondiagnostic ECG warrants heightened awareness and close inspection to ensure accurate analysis.

## Figures and Tables

**Figure 1:**
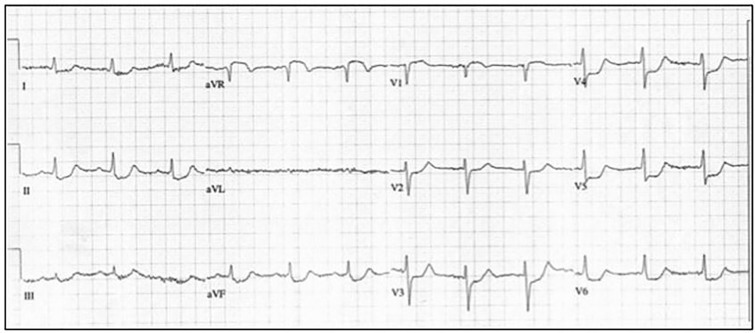
The Forgotten Lead. Diffuse ST depression with ST elevation in aVR>1mm and subtle ST elevation in V1; ST elevation in aVR>V1.

**Figure 2:**
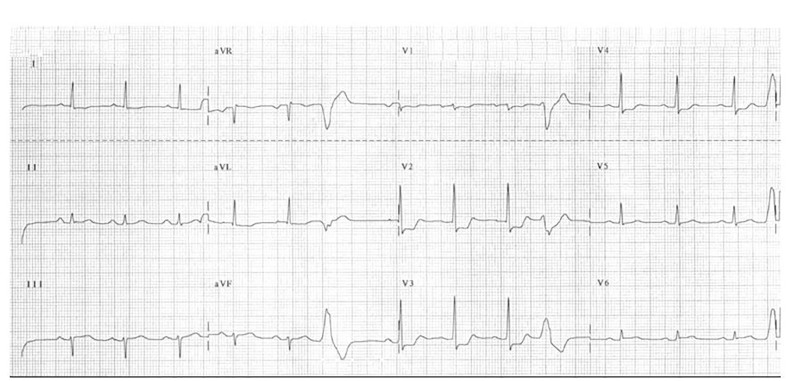
Posterior acute myocardial infarction (AMI). Anteroseptal (V1–V3/4) ST depression with tall R waves and upright T waves.

**Figure 3:**
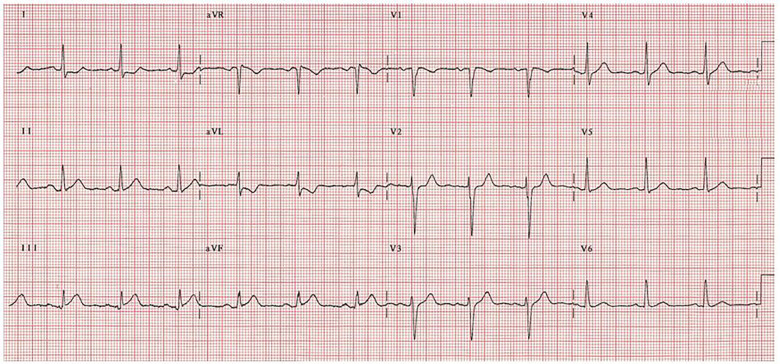
Inferior AMI. High lateral (I, aVL) ST depression with inferior (II, III, aVF) ST elevation.
